# *Blastocystis* spp. subtype 10 infected beef cattle in Kamal and Socah, Bangkalan, Madura, Indonesia

**DOI:** 10.14202/vetworld.2020.231-237

**Published:** 2020-02-07

**Authors:** Lucia Tri Suwanti, Yuli Susana, Poedji Hastutiek, Endang Suprihati, Nunuk Dyah Retno Lastuti

**Affiliations:** 1 Department of Veterinary Parasitology, Faculty of Veterinary Medicine, Universitas Airlangga, Jl. Mulyorejo, Kampus C Unair, Surabaya, 60115, Indonesia; 2 Toxoplasmosis Study Group, Institute of Tropical Diseases, Universitas Airlangga, Jl. Mulyorejo, Campus C Unair, Surabaya 60115, Indonesia; 3 Magister Student, Faculty of Veterinary Medicine, Universitas Airlangga, Jl. Mulyorejo, Campus C Unair, Surabaya 60115, Indonesia

**Keywords:** beef cattle, *Blastocystis* spp. subtype 10, Madura, Indonesia, zoonotic

## Abstract

**Background and Aim:**

*Blastocystis* spp. is a gastrointestinal parasite that can infect both humans and animals and has the potential to become a zoonotic parasite. This study analyzed a subtype (ST) of *Blastocystis* spp. that had infected beef cattle in Kamal and Socah, Bangkalan, Madura, Indonesia.

**Materials and Methods:**

Fresh stool samples were collected from 108 beef cattle at Kamal and Socah, Bangkalan, Madura, Indonesia. *Blastocystis* spp. were detected both morphologically and genetically based on the 18S rRNA gene. The morphology of *Blastocystis* spp. from the stool samples and cultured samples were observed under a light microscope. *Blastocystis* spp. from 20 positive cultures were amplified through polymerase chain reaction, and the resultant sequences were identified by ST.

**Results:**

One hundred and eight (100%) fecal samples from the fresh or cultured stools were positive morphologically for *Blastocystis* spp. Molecularly, all 20 of the samples selected for DNA analysis were found to be *Blastocystis* spp. ST 10.

**Conclusion:**

Based on morphological and molecular detection, the prevalence of *Blastocystis* spp. infection in beef cattle within Kamal and Socah, Bangkalan, Madura, Indonesia, was high. About 100% were non-zoonotic parasites. This was the first report of *Blastocystis* spp. ST 10 found in infected beef cattle in Kamal and Socah, Bangkalan, Madura, Indonesia.

## Introduction

Madura Island, including the Bangkalan district, is known as a beef cattle production area. The population of beef cattle in Bangkalan was 200,279 heads in 2017 with 5627 heads spread out in the Kamal subdistrict [1]. According to Hariyono *et al*. [2], beef cattle farming on Madura Island is still traditionally managed. Production of cattle is generally a side business, and the educational background of the farmer is often insufficient terminating at the elementary school level [3]. Thus, inadequate management systems correlate to a high disease burden in livestock, including parasitic diseases [4].

*Blastocystis* spp . is a gastrointestinal parasite found in feces samples from both humans and animals [5], including mammals, birds, amphibians, and reptiles [6]. Several studies have shown that *Blastocystis* infection has the potential to be a zoonotic parasite, as evidenced by the discovery of the same subtype (ST) in both humans and animals [7]. *Blastocystis* is distributed worldwide with varying prevalence in many countries. The prevalence of *Blastocystis* infection in humans within developing countries is significantly higher (50%) than that found in established countries (20%) [8,9]. Furthermore, *Blastocystis* cases in children from Senegal can reach 100% [10]. This difference is due to poor hygiene practices, close animal contact, and consumption of contaminated food or water [11]. *Blastocystis* infection is a waterborne or foodborne disease, and transmission occurs through the fecal–oral route for the infective stage of the cyst form [12,13]. *Blastocystis* isolates from humans are referred to as *Blastocystis hominis* and isolates from animals are generally referred to as *Blastocystis* spp. Further, classification is often based on the type of host [14]. In general, the way to detect *Blastocystis* spp. in the feces is with a direct smear examination using a light microscope or by *in vitro* culture. *Blastocystis* spp. isolates from humans and other animals exhibit similar morphology. There are four morphological forms of Blastocystis including vacuolar, granular, amoeboid, or cyst forms [11]. Polymerase chain reaction (PCR) is the most sensitive method for the diagnosis of this parasite [14]. Many researchers have identified *Blastocystis* in animals globally. Cattle in Iran were found to be infected with *Blastocystis* ST 3, 5, and 6 as well as unidentified STs [14]. Furthermore, non-primate animal species in a wildlife park in the UK were found to be positive with six STs: ST1, ST4, ST5, ST10, ST14, and a potentially new ST [15]. In Brazil, from a total of 334 stool samples, 28 different genera of animals were found in six STs: ST1, ST2, ST3, ST4, ST5, and ST8 [16]. However, information about the prevalence of *Blastocystis* infection in various animals in Indonesia is still very limited. Yoshikawa *et al*. [17] surveyed *Blastocystis* STs from humans, domestic pigs, domestic chickens, and wild rodents on Sumba Island, Indonesia, and they found STS 1-3 in children and ST5, ST7, and ST4 in domestic pigs, chickens, and wild rodents, respectively.

Our previous study found that *Blastocystis* infection in beef cattle within Bangkalan, Madura, Indonesia, was based on the morphology of the organism [18]. Our previous research also found *Blastocystis* that was morphologically identified in fresh and cultured stool samples from the sugar glider [19].

This study aimed to assess the molecular characteristics of *Blastocystis* spp. found in beef cattle at Kamal and Socah, Madura. Knowledge of the exact ST of *Blastocystis* infecting these cities is paramount to design a management program to control this parasite.

## Materials and Methods

### Ethical approval

Ethical approval for this study was obtained from the Animal Care and Use Committee of the Faculty of Veterinary Medicine, Universitas Airlangga (approval number: 1.KE. 063.01.2018).

### Study period, location and sample collection

This study was conducted between July 2018 and August 2018. Sampling was conducted on July 11, 2018, on beef cattle farms at Kamal and Socah subdistrict, Bangkalan district, Madura. The number of samples was 108 (56 samples from Kamal and 52 from Socah) from 55 farms, and the subject cattle ranged from 0.5 to 7 years of age. The location of farms was in close proximity to the community. The samples consisted of fresh stool collected and stored in labeled sterile containers and then transported incold condition (8^o^C) to the Department of Veterinary Parasitology, Faculty of Veterinary Medicine, Universitas Airlangga.

### Microscopic examination

Parasitological observations were carried out at the Laboratory of the Department of Veterinary Parasitology, Faculty of Veterinary Medicine, Airlangga University. A small amount of feces was diluted with water (1:9) and then was filtered. The fecal sample solution was then centrifuged at 1500 rpm for 5 min, the supernatant was discharged, and the pellets of each sample were smeared onto three glass slides. The first was used to observe the specimen, the second and the third involved the addition of iodine or Giemsa solution around 50 µl (2-3 drops), respectively. Observations were conducted under light microscope 100-400×.

### *In vitro* culture

Approximately 1 g of each fecal sample was inoculated into a sterile conical tube containing 1 ml of simple medium. Each sample was incubated at 37°C for 72 h. The simple medium [6] was comprised 500 ml Ringer’s solution (Otsu-RL^®^ Otsuka, Indonesia), 0.5 g yeast extract (Merck, Germany), 5 g peptone (Merck, Germany), 20 ml boiled rice water, and 50-100 mg oxytetracycline (Vet-oxy LA, Sanbe, Indonesia.). Following 72 h of incubation, the culture was examined for the presence and growth of *Blastocystis*. Briefly, a little supernatant just above the sediment of the stool was taken and dropped onto a glass object and observed under a light microscope. Positive samples were subsequently maintained by subculturing, and *Blastocystis* spp. were harvested by removing the culture medium supernatant, which was then centrifuged at 1500 rpm for 10 min. Pellets were removed and resuspended in 1 ml phosphate-buffered saline and stored at −20°C for PCR.

### DNA extraction and amplification

Then, 20 positive culture samples (10 samples from Kamal and 10 from Socah) were selected for DNA extraction and amplification. DNA extraction was performed using the QIAamp DNA Stool Mini Kit (QIAGEN, Hilden, Germany) according to the manufacturer’s protocol. Then, the DNA samples were stored at −20°C for later use. *Blastocystis*-specific primers b11400 FORC (5`-GGA ATC CTC TTA GAG GGA CAC TAT ACA T-3`) and b11710 REVC (5`-TTA CTA AAA TCC AAA GTG TTC ATC GGA C-3) were used for amplification of the DNA [14]. The PCR was conducted in a thermocycler (Bioer Genetouch Thermal Cycler TC-E-48DA, China) using the following conditions initial denaturing at 94°C for 5 min, followed by 35 cycles of 94°C for 30 s, 50°C for 30 s, 72°C for 30 s, and finally 1 cycle of 72°C for 5 min. The amplification products were then electrophoresed using 1.5% agarose gels (Thermo Scientific, USA) in Tris-borate-EDTA buffer. Gels were stained with DNA gel stain (RedSafe^™^, Intron Biotechnology, Korea) and visualized using an ultraviolet gel documentation system (ATTO AE-33FXN Printgraph, Japan). The DNA fragment size was estimated using a 100 bp ladder (Promega, USA) and the expected PCR product was 310 bp. The primer F and R positions were 1344-1371 and 1659-1632, respectively. *Blastocystis hominis* isolate RU8 small ribosomal RNA gene subunit, partial sequence (accession no. KF002562) [9], and the reference sequence was GGAATCCTCTTAGAGGGACACTA TACATAAAGTATAGGGAAGCTGGAGGCAATA ACAGGTCTGTGATGCCCTTAGATGTCCTGGG CTGCACGCGCGCGACACTGATTCATTCAAC AAGTGGCTGAATCGATAGATTTGGCAA ATCTTTTGAAAATGAATCGTGATGGGG ATTGATGTCTGTAATAAACGTCATGAACGA GGAATTCCTAGTAAATGCAAGTCATCAACT TGCGTTGATTACGTCCCTGCCCTTTGTA CACACCGCCCGTCGCACCTACCGATTGA ATGGTCCGATGAACACTT TG GA TT TT AGTAA.

Then, each of the positive samples was amplified using seven pairs of STs primers (ST1-7) to determine the ST of *Blastocystis*. This protocol followed the procedure manual from the manufacturer. The DNA from the PCR products with 310 bp was sequenced and then seven pairs of STS primers (ST1-7) were used to identify STs of *Blastocystis* spp.

### DNA sequencing and phylogenetic tree analyzing

All PCR products at 310 bp were sent to the First BASE Sdn Bhd Laboratories (Malaysia) through PT. Genetika Science (Indonesia) to be sequenced. All of the nucleotide sequences were aligned using the BioEdit sequence alignment editor (https://bioedit.software.informer.com/download/). Nucleotide sequences were compared to the obtained sequences in the GenBank database using the Basic Local Alignment Search Tool to determine the isolates of *Blastocystis* spp. (http://www.blast.ncbi.nlm.nih.gov/Blast.cgi). The phylogenetic tree was constructed using neighbor-joining (NJ). Analysis of the 18S ssrRNA for *Blastocystis* spp. was conducted with computer software Molecular Evolutionary Genetic Analysis (MEGA) version 7 (http://www.megasoftware.net).

### Statistical analysis

The prevalence of *Blastocystis* infection in beef cattle in Kamal and Socah was analyzed through the Chi-square test using SPSS version 20.0, (IBM SPSS Statistics, IBM Corporation, New York, USA). p<0.05 was regarded as statistically significant.

## Results

The total number of samples was 108 collected from 55 farms of beef cattle. The educational background of most of the farmers was graduated from elementary school. One farmer had 2-5 cattle caged in one small place. The cages were located close to the farmers’ houses. Almost all of the cage floors were soil, and living conditions were dirty, feces piled up in the cage. Grass was the main feed for the cattle in this area, with no existing feedlots.

**Figure-1:**
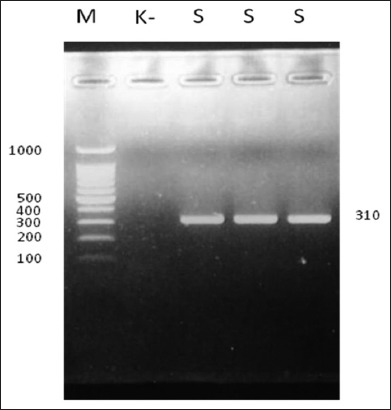
Polymerase chain reaction of DNA for *Blastocystis spp*. from cattle in Kamal and Socah, Madura. M=DNA leader, K−=Negative control, S=Sample.

All of the 108 beef cattle samples (100%) were infected with *Blastocystis* spp., based on the morphology observations of the fresh stool (wet mount) samples and the *in vitro* cultures (Table-1). Twenty samples were selected for further analysis using DNA. The *in vitro* cultures were confirmed for the presence of *Blastocystis* by amplifying the DNA with primers specific to *Blastocystis* spp. and then all of the samples that were molecularly positive were indicated with a band (approximate 310 bp) in the agarose gel (Figure-1); furthermore, seven pairs of STS primers (ST1-7) were used to detect STs in the DNA which could not be detected by the agarose gel electrophoresis.

**Table-1:** Prevalence of *Blastocystis* infection in the examined feces of beef cattle in Bangkalan, Madura.

Subdistrict	Wet mount number positive/number of sample (%)	Culture number positive/number of Sample (%)	PCR number positive/number of sample (%)
Kamal	56/56 (100)	56/56 (100)	10/10 (100)
Socah	52/52 (100)	52/52 (100)	10/10 (100)
Total	108/108 (100)	108/108 (100)	20/20 (100)

PCR=Polymerase chain reaction

Using forward and reverse primer, the PCR purified products of both samples from Kamal and Socah were successfully sequenced to identify an ST of *Blastocystis* spp. The sequences for the 20 selected samples were matched with a partial sequence 18S ssrRNA gene of *Blastocystis* spp. ST 10 isolate CA6 under accession no. KC148207.1. Values for maximal score, total score, Corey cover, and percentage identity were 466-523, 466-523, 92-97%, and 97.39-98.52%, respectively (Table-2).

**Table-2:** Basic local alignment search tool sequence DNA of *Blastocystis* spp. in beef cattle from Bangkalan, Madura, compared with the published data on gene bank, partial sequences of 18S ssrRNA genes for *Blastocystis spp.* ST 10 isolate CA6 under accession no. KC148207.1.

Sample	Characteristics of cattle			Accession number of sample	Accession number reference	Maximal score	Total score	Corey cover (%)	Identity (%)

	Sex	Age	Healthy status						
Kamal 1	Female	7 years	No diarrhea	MN606117	KC148207.1	473	473	92	98.51
Kamal 2	Male	3 years	Diarrhea	MN606118	KC148207.1	477	477	92	98.52
Kamal 3	Male	10 months	Diarrhea	MN606119	KC148207.1	477	477	93	98.18
Kamal 4	Male	1.5 years	No diarrhea	MN606120	KC148207.1	477	477	93	98.18
Kamal 5	Male	3 years	No diarrhea	MN606121	KC148207.1	475	475	93	98.52
Kamal 6	Male	6 months	No diarrhea	MN606122	KC148207.1	477	477	93	98.52
Kamal 7	Male	1.5 years	No diarrhea	MN606123	KC148207.1	477	477	93	98.52
Kamal 8	Male	2.5 years	Diarrhea	MN606124	KC148207.1	466	466	94	97.45
Kamal 9	Female	5 years	No diarrhea	MN606125	KC148207.1	473	473	92	98.51
Kamal 10	Female	6 years	No diarrhea	MN606126	KC148207.1	479	479	94	98.18
Socah 1	Female	1 year	No diarrhea	MN606127	KC148207.1	472	472	93	97.81
Socah 2	Female	1 year	No diarrhea	MN606128	KC148207.1	472	472	93	97.81
Socah 3	Male	7 months	No diarrhea	MN606129	KC148207.1	479	479	93	98.18
Socah 4	Female	7 years	No diarrhea	MN606130	KC148207.1	475	475	93	98.52
Socah 5	Female	3 years	Diarrhea	MN606131	KC148207.1	477	477	93	98.52
Socah 6	Female	3 years	No diarrhea	MN606132	KC148207.1	472	472	94	97.82
Socah 7	Male	2 years	No diarrhea	MN606133	KC148207.1	477	477	92	98.52
Socah 8	Female	5 months	Diarrhea	MN606134	KC148207.1	479	479	93	98.18
Socah 9	Female	1 year	No diarrhea	MN606135	KC148207.1	472	472	93	98.52
Socah 10	Female	7 years	Diarrhea	MN606136	KC148207.1	523	523	97	97.39

**Figure-2:**
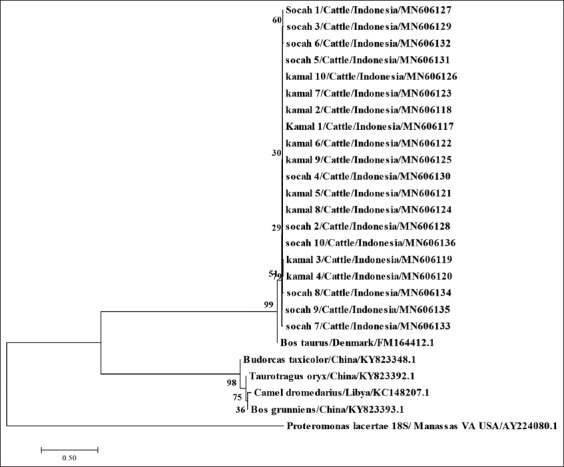
Evolutionary relationships for taxa of *Blastocystis* spp. ST 10. The evolutionary history was inferred using the neighbor-joining method (bootstrap 1000). The optimal tree with the sum of branch length=2.13564045 is shown (next to the branches). The evolutionary distances were computed using the maximum composite likelihood method and are in the units of the number of base substitutions per site. This analysis involved 28 nucleotide sequences. Codon positions included were 1^st^, 2^nd^, and 3^rd^ non-coding. All positions with gaps or missing data were eliminated. There were a total of 241 positions in the final dataset. Evolutionary analyses were conducted in Molecular Evolutionary Genetic Analysis 7.

The position of *Blastocystis* spp. from Bangkalan Madura on the phylogenetic tree based on the SSU rRNA gene sequence for *Blastocystis* spp. ST 10 is shown in Figure-2. The rooted NJ tree identified only one clade that corresponded to *Blastocystis* ST 10 in *Bos taurus* from Denmark (FM164412).

## Discussion

The prevalence of *Blastocystis* infection in beef cattle in Kamal and Socah, Bangkalan, Madura, was extremely high, 100%. This condition is related to the management of the farms in Kamal and Socah, especially in terms of the cleanliness of the cages and housing systems. According to Welay *et al*. [4], insufficient management systems are correlated with a high disease burden in livestock. *Blastocystis* infection is a waterborne or foodborne disease transmitted by the fecal-oral route [11-13]. When cattle are kept in the same cage, transmission occurs between the cows. Moreover, the cage floor is ground, so the conditions are dirty with no place to graze for food.

Compared to epidemiological studies from several other countries, the prevalence of *Blastocystis* spp. infection in cattle in Kamal and Socah, Madura, was the highest at 100%. Research conducted in other countries found the following results for the prevalence of 1.8% in Spain [20], 19.15% in the USA [21], 34.5% in Malaysia [22], 9.6% in Iran [14], 9.6% in China [23], and 50% in Thailand [24]. Our previous study showed that the prevalence of *Blastocyst* infections in sugar gliders from Surabaya was also very high (100%) [19]. Yoshikawa *et al*. [17] reported that the prevalence of *Blastocyst* infections in animals from Sumba, Indonesia, depended on the genus of the animals such as pigs, chickens, or wild rodents and the results were 87.1%, 34.2%, and 13.0%, respectively.

There are four distinct morphological forms for *Blastocystis* spp. including vacuolar, granular, amoeboid, and cyst forms [11]. Since these parasites are pleomorphic organisms, they can be confused with other organisms, in fecal samples in the absence of staining [25]. Therefore, this study used wet mount, iodine, and Giemsa staining. Our study failed to find the amoeboid form, while the other three forms, vacuolar, granular, and cyst, were isolated. However, this was an expected result as Ahmed and Karanis [26] found that the amoeboid form is rarely reported.

PCR is the most sensitive method for *Blastocystis* diagnosis (14). According to Badparva *et al*. [14], using the primers b11400 FORC and b11710 REVC, the DNA sequence size for the genus *Blastocystis* was 310 bp, which was in accordance with the results of this study, and convinced us that the organism found in the cattle feces samples was a genus of *Blastocystis*. In the current study, all of the 20 DNA samples (100%) were positive for this parasite. However, this study failed to determine *Blastocystis* ST using the STS primers (St1-7). This suggested that *Blastocystis* ST from Kamal and Socah did not belong to *Blastocystis* STs 1-7. Furthermore, this research did not use barcoded regions to detect the ST because extraction of the DNA was conducted using cultures of *Blastocystis*. This was because, according to research conducted by Stensvold [27], the barcoding PCR had higher applicability and sensitivity for fresh direct stool samples, while the STs primers generally worked better for DNA extracted from *Blastocystis* cultures.

Since the STs primer could identify STs of *Blastocystis* samples, we sequenced the PCR product. All of 20 selected samples were confirmed to be *Blastocystis* spp. ST10. The similarity percentages for the isolated *Blastocystis* spp. ST10 CA6 (accession no. KC148207.1) sequences were 97.39-98.52%. The results in the present study were highly similar to research by Santin *et al*. [28] that reported that their cattle samples were also only infected with ST10. *Blastocystis* spp. ST10 was also found in several other countries, and it was a predominant ST in cattle from Denmark [29], the USA [21], the UK, Libya [30], in China [23], in Thailand [28], and in Lebanon [31]. Therefore, the findings of this research reinforced the hypothesis by Cian *et al*. [5] that *Bovidae* may be natural hosts of *Blastocystis* spp. ST10. Their research showed that various *Bovidae* in a French Zoo were infected by ST10 and ST14. In Indonesia, Yoshikawa *et al*. [17] reported that a pig, chicken, and wild rodent in Sumba were infected with *Blastocystis* ST 1, 2, 4, and 7. This is the first report of *Blastocystis* ST 10 infection in cattle from Indonesia.

All of 20 DNA samples were located in the same clade with *Blastocystis* ST 10 from *B. taurus* from Denmark (FM164412). However, this was a different clade of *Blastocystis* ST 10 from other ruminants, including *Budorcas taxicolor* from China (KY823348.1), *Taurotragus* *oryx* from China (KY823348.1), *Camelus dromedarius* from Libya (KC148207.1), and *Bos grunniens* from China (KY823393.1).

Although *Blastocystis* spp. ST10 is not considered a zoonotic organism, the existence of a high prevalence of ST10 in Bangkalan, Madura, in such close proximity to humans creates a potential for future transmission. Moreover, it poses a risk for other types of livestock and pets in Madura as a potential source of transmission. Domesticated animals that have already been reported to be infected by ST10 included sheep in China [32] and goats in Thailand [24], China [33], and Turkey [34]. Noradilah *et al*. [35] also reported *Blastocystis* spp. ST10 in dogs, goats, and birds in Malaysia.

## Conclusion

Beef cattle in Kamal and Socah, Bangkalan, Madura, Indonesia, were infected by *Blastocystis* spp. ST10. This finding added to the literature that *Blastocystis* ST 10 is a predominant ST in cattle and it was the first report of *Blastocystis* ST 10 infection in cattle in Indonesia. Moreover, the prevalence found in this study was significantly higher than in other countries.

## Authors’ Contributions

LTS, YS, ES, PH, and NDRL designed the concept for this research and scientific paper. All authors conducted the research. LTS and YS collected samples from the fields. LTS, YS, and NDRL conducted the laboratory work. All authors participated in the draft and revision of the manuscript and approved the final manuscript.

## References

[1] Department of Communication and Information Bangkalan Regency (2017). Bangkalan Regional Statistics.

[2] Hariyono M.B, Hartutik A, dan Andayani S.D (2010). Economic potential of raising livestock in the area of post-Suramadu Madura. J. Ternak Trop.

[3] Rahmawati S.A, Harijani N, Lamid M (2015). Analysis of income of Madura cattle farmers and Madrasin cattle in Taman Sareh village, Sampang sub-district. J. Agro. Vet.

[4] Welay G.M, Tedla D.G, Teklu G.G, Weldearegay S.K, Shibeshi M.B, Kidane H.H, Gebrezgiabher B.B, Abraha T.H (2018). A preliminary survey of major diseases of ruminants and management practices in Western Tigray province, Northern Ethiopia. BMC Vet. Res.

[5] Cian A, El Safadi D, Osman M, Moriniere R, Gantois N, Benamrouz-Vanneste S, Delgado-Viscogliosi P, Guyot K, Li L.L, Monchy S, Noel C, Poirier P, Nourrisson C, Wawrzyniak I, Delbac F, Bosc S, Chabe M, Petit T, Certad G, Viscoglios E (2017). Molecular epidemiology of *Blastocystis* spp. in various animal groups from two French zoos and evaluation of the potential zoonotic risk. PLoS One.

[6] Mohammed S.T, Sulaiman N.M, Kamal S.B (2015). Preparation of simplified culture for culturing *Blastocystis hominis* Parasite. J. Biol. Agri. Healthc.

[7] Osman M, El Safadi D, Cian A, Benamrouz S, Nourrisson C, Poirier P, Pereira B, Razakandrainibe R, Pinon A, Lambert C, Wawrzyniak I, Dabboussi F, Delbac F, Favennec L, Hamze M, Viscogliosi E, Certad G (2016). Prevalence and risk factors for intestinal protozoan infections with *Cryptosporidium*. *Giardia*
*Blastocystis* and *Dientamoeba* among school children in Tripoli, Lebanon. PLoS Negl. Trop. Dis.

[8] El Safadi D, Cian A, Nourrisson C, Pereira B, Morelle C, Bastien P, Bellanger A.P, Boterel F, Candolfi E, Desoubeaux G, Lachaud L, Morio F, Pomares C, Rabodonirina M, Wawrzyniak I, Delbac F, Gantois N, Certad G, Delhaes L, Poirier P, Viscogliosi E (2016). Prevalence, risk factors for infection and subtype distribution of the intestinal parasite *Blastocystis* spp. from a large-scale multi-center study in France. BMC Infect. Dis.

[9] Ramirez J.D, Sanchez L.V, Bautista D.C, Corredor A.F, Florez A.C, Stensvold C.R (2014). Blastocyst is subtypes detected in humans and animals from Colombia. Infect. Genet. Evol.

[10] El Safadi D, Gaayeb L, Meloni D, Cian A, Poirier P, Wawrzyniak I, Delhaes L, Seck M, Hamze M, Riveau G, Viscogliosi E (2014). Children of Senegal river basin show the highest prevalence of *Blastocystis* spp. ever observed Worldwide. BMC Infect. Dis.

[11] Wawrzyniak I, Poirier P, Viscogliosi E, Dionigia M, Texier C, Delbac F, El Alaoui H (2013). *Blastocystis* an unrecognized parasite:An overview of pathogenesis and diagnosis. Adv. Infect. Dis.

[12] Yoshikawa H, Abe N, Wu Z (2004). PCR-based identification of zoonotic isolates of *Blastocystis* from mammals and birds. Microbiology.

[13] Lee L, Chye T.T, Karmacharya B.M, Govind S.K (2012). *Blastocystis* spp.:Waterborne zoonotic organism, a possibility?. Parasit Vectors.

[14] Badparva I.R, Sadraee J, Kheirandish F (2015). Genetic diversity of *Blastocystis* isolated from cattle in Khorramabad. Jundishapur J. Microbiol.

[15] Betts E.L, Gentekaki E, Thomasz A, Breakell V, Carpenter A.I, Tsaousis A.D (2018). Genetic diversity of *Blastocystis* in non-primate animals. Parasitology.

[16] Valença-Barbosa C, do Bomfim T.C.B, Teixeira B.R, Gentile R, Neto S.F.C, Magalhães B.S.N, Balthazar D.A, Silva F.A, Levy C.M.A, Santos H.L.C (2019). Molecular epidemiology of *Blastocystis* isolated from animals in the State of Rio de Janeiro, Brazil. PLoS One.

[17] Yoshikawa H, Tokoro M, Nagamoto T, Arayama S, Asih P.B, Rozi E, Syafrudi D (2016). Molecular survey of *Blastocystis* spp. from humans and associated animals in an Indonesian community with poor hygiene. Parasitol. Int.

[18] Hastutiek P, Yuniarti W.M, Djaeri M, Lastuti N.D.R, Suprihati E, Suwanti L.T (2019). Prevalence and diversity of gastrointestinal protozoa in Madura cattle at Bangkalan regency, East Java, Indonesia. Vet. World.

[19] Natalia N, Suwanti L.T, Suprihati E, Kusnoto Koesdarto S, Srianto P (2018). Morphological detection of the intestinal parasite *Blastocystis* spp. in fresh and cultured feces of pet sugar gliders (*Petaurus breviceps*) (*Mammalia*. *Marsupialia*. *Petauridae*) in Surabaya, Indonesia *Philipp*. J. Vet. Med.

[20] Quilez J, Sanchez-Acedo C, Clavel A, Causape A.C (1995). Occurrence of *Blastocystis* spp. in Cattle in Aragón, Northeastern Spain. Parasitol. Res.

[21] Fayer R, Santin M, Macarisin D (2012). Detection of concurrent infection of dairy cattle with *Blastocystis Cryptosporidium Giardia* and *Enterocytozoon* by molecular and microscopic methods. Parasitol. Res.

[22] Hemalatha C, Chandrawathani P, Kumar G.S, Premaalatha B, Geethamalar S, Rozita M.H.L, Haziqah M.T.F, Sabapathy D, Ramlan M (2014). The Diagnosis of *Blastocystis* spp. from animals an emerging zoonosis. Malays. J. Vet. Res.

[23] Zhu W, Tao W, Gong B, Yang H, Li Y, Song M, Lu Y, Li W (2017). First report of *Blastocystis* infections in cattle in China. Vet. Parasitol.

[24] Udonsom R, Prasertbun R, Mahittikom A, Mori H, Changbunjong T, Komalamisra C, Pintong A.R, Sukthana Y, Popruk S (2018). *Blastocystis* infection and subtype distribution in humans, cattle, goats, and pigs in Central and Western Thailand. Infect. Genet. Evol.

[25] Bergamo do Bomfim T.C, Machado do Couto M.C (2013). Morphological diagnosis and occurrence of *Blastocystis* spp. obtained from the stool samples of domestic bird species commercialized in municipal markets. J. Parasitol. Vector Biol.

[26] Ahmed S.A, Karanis P (2018). *Blastocystis* spp., ubiquitous parasite of humans, animals and the environment. In:Encyclopedia of Environmental Health.

[27] Stensvold C.R (2013). Comparison of sequencing (barcode region) and sequence-tagged-site PCR for *Blastocystis* subtyping. J. Clin. Microbiol.

[28] Santin M, Gomez-Munoz M.T, Solano-Aguilar G, Fayer R (2011). Development of a new PCR protocol to detect and subtype *Blastocystis* spp. from humans and animals. Parasitol. Res.

[29] Stensvold C.R, Alfellani M.A, Norskov-Lauritsen S, Prip K, Victory E.L, Maddox C, Nielson H.V, Clark C.G (2009). Subtype distribution of *Blastocystis* isolates from synanthropic and zoo animals and identification of a new subtype. Int. J. Parasitol.

[30] Alfellani M.A, Taner-Mulla D, Jacob A.S, Imeede C.A, Yoshikawa H, Stensvold C.R, Clark C.G (2013). Genetic diversity of *Blastocystis* in livestock and zoo animals. Protist.

[31] Greige S, El-Safadi D, Khaled S, Gantois N, Baydouna B, Chemalyc M, Benamrouz-Vannestea S, Chabea M, Osman M, Certada G, Hamzeb M, Viscogliosia E (2019). First report on the prevalence and subtype distribution of *Blastocystis* spp. in dairy cattle in Lebanon and assessment of zoonotic transmission. Acta Trop.

[32] Li W.C, Wang K, Gu Y (2018). Occurrence of *Blastocystis* spp. and *Pentatrichomonas hominis* in sheep and goats in China. Parasit. Vectors.

[33] Song J.K, Yin Y.L, Yuan Y.J, Tang H, Ren G.J, Zhang H.J, Li Z.X, Zhang Y.M, Zhao G.H (2017). First genotyping of *Blastocystis* spp. in dairy, meat, and cashmere goats in Northwestern China. Acta Trop.

[34] Aynur Z.E, Guclu O, Yildiz I, Aynur H, Ertablakar H, Bozdogan B, Ertug S (2019). Molecular characterization of *Blastocystis* in cattle in Turkey. Parasit. Res.

[35] Noradilah S.A, Anuar T.S, Moktar N, Lee I.L, Salleh F.M, Manap S.N.A, Mohtar N.S.H, Azrul S.M, Abdullah W.O, Nordin A, Abdullah S.R (2017). Molecular epidemiology of *Blastocystis* spp. in animals reared by the aborigines during wet and dry seasons in rural communities, Pahang, Malaysia Southeast. Asian J. Trop. Med. Public Health.

